# Bioactive-Enriched Chitosan/Poly(vinyl Alcohol) Electrospun Nanofibers for Wound Healing: In Vitro and In Vivo Evaluation

**DOI:** 10.3390/ph19040581

**Published:** 2026-04-05

**Authors:** Teodora Iurascu, Andreea-Teodora Iacob, Carmen Solcan, Cristina Mariana Uritu, Bianca-Stefania Profire, Narcisa Laura Marangoci, Adina Coroaba, Andrei Szilagyi, Ivona Costachescu, Maria-Raluca Gogu, Leontina-Elena Filipiuc, Lenuta Profire

**Affiliations:** 1 Department of Pharmaceutical and Therapeutic Chemistry, Faculty of Pharmacy, Grigore T. Popa University of Medicine and Pharmacy Iasi, 16 University Street, 700115 Iasi, Romania; teodora-l-hanghicel@d.umfiasi.ro (T.I.); lenuta.profire@umfiasi.ro (L.P.); 2 Faculty of Veterinary Medicine, Ion Ionescu de la Brad University of Life Sciences, 3 Mihail Sadoveanu Alley, 700490 Iasi, Romania; carmen.solcan@iuls.ro; 3 Centre of Advanced Research in Bionanoconjugates and Biopolymers, Petru Poni Institute of Macromolecular Chemistry, 41A Grigore Ghica Voda Alley, 700487 Iasi, Romania; cristina-mariana.uritu@umfiasi.ro (C.M.U.); nmarangoci@icmpp.ro (N.L.M.); adina.coroaba@icmpp.ro (A.C.); 4 Advanced Center for Research and Development in Experimental Medicine “Prof. Ostin C. Mungiu”, Grigore T. Popa University of Medicine and Pharmacy Iasi, 700115 Iasi, Romania; andrei.szilagyi@umfiasi.ro (A.S.); ivona.costachescu@umfiasi.ro (I.C.); raluca.gogu@umfiasi.ro (M.-R.G.); leontina.filipiuc@umfiasi.ro (L.-E.F.); 5 Department of Internal Medicine, Faculty of Medicine, Grigore T. Popa University of Medicine and Pharmacy Iasi, 16 University Street, 700115 Iasi, Romania; bianca-stefania.profire@umfiasi.ro

**Keywords:** hemocompatibility, oxidative stress, angiogenesis, wound healing

## Abstract

**Background:** Wound healing remains a major clinical challenge, often impaired by persistent inflammation, oxidative stress, and abnormal extracellular matrix remodeling. Electrospun nanofibers (NFs) have emerged as promising wound dressing platforms due to their biomimetic structure and capacity to incorporate multiple bioactive compounds (ACs) with synergistic therapeutic effects. **Objectives:** This study aimed to biologically assess novel chitosan/poly(vinyl alcohol) (CH/PVA) NFs functionalized with natural active compounds (L-arginine—ARG, allantoin—ALA, royal jelly—RJ, and curcumin—CUR) as multifunctional systems for wound healing and tissue remodeling. **Methods:** The nanofibrous systems performed the in vitro evaluation of antioxidant activity (DPPH, ABTS, FRAP, PRAP), anti-inflammatory potential (protein denaturation test), hemocompatibility, and cytocompatibility using dermal fibroblasts. In vivo healing performance was evaluated in an excisional wound model using macroscopic wound contraction analysis, histopathology, and immunohistochemical staining (MMP-9, CD31, VEGF-A, α-SMA). **Results:** The bioactive-enriched CH/PVA NFs exhibited strong antioxidant and anti-inflammatory activity, excellent hemocompatibility (hemolysis < 5%), and excellent cytocompatibility, with promoting fibroblast proliferation. In vivo experiments revealed that the treated groups exhibited accelerated wound closure, improved re-epithelialization, increased angiogenesis, and showed more efficient tissue remodeling compared to the controls, as validated by histological and immunohistochemical studies. **Conclusions:** The findings indicate that bioactive-enriched CH/PVA NFs serve as effective, biocompatible, and multifunctional matrices for wound healing, hence endorsing their potential for further translational advancement in skin regeneration applications.

## 1. Introduction

Delayed wound healing represents a persistent clinical challenge, frequently associated with bacterial infections and prolonged inflammation. These diseased circumstances significantly impact essential biological processes related to tissue healing, including proteolytic enzyme activity, oxidative stress control, angiogenesis, plasminogen activator synthesis, and the accumulation of cytotoxic metabolites [1]. Thus, treatment approaches that concurrently restrict microbial colonization and regulate the inflammatory response are crucial for facilitating effective wound healing.

Recently, natural active compounds (ACs) have gained recognition as effective therapeutic agents in wound treatment due to their extensive antibacterial, antioxidant, anti-inflammatory, and regenerative effects, together with their superior biocompatibility and safety profiles [2,3,4]. Curcumin [5,6], allantoin [7], L-arginine [8,9], and royal jelly [10,11] have attracted significant attention due to their synergistic mechanisms of action and established effectiveness in promoting tissue healing, being widely explored as functional additives in a range of biomaterial platforms.

Curcumin (CUR) is a polyphenolic compound recognized for its significant anti-inflammatory and antioxidant effects, often shown to improve wound healing and facilitate tissue remodeling by modulating inflammatory signaling pathways and diminishing oxidative stress [1,12]. CUR has been successfully incorporated into hydrogels, membranes, and composite scaffolds, where it has demonstrated pronounced antioxidant, anti-inflammatory, and pro-angiogenic activities relevant to wound healing [13,14].

Allantoin (ALA) demonstrates antioxidant, anti-inflammatory, keratolytic, and moisturizing properties while promoting cell proliferation and enhancing extracellular matrix (ECM) hydration. By enhancing fibroblast activity and extracellular matrix formation (ECM), ALA facilitates accelerated tissue remodeling and wound closure [15]. ALA has been utilized in gelatin- and alginate-based scaffolds and hydrogels, exhibiting excellent cytocompatibility and promoting tissue regeneration processes [16].

L-arginine (ARG) functions in wound healing as a precursor for proline through arginase-mediated metabolism, which is vital for collagen synthesis, and by producing nitric oxide (NO) via oxidative deamination, a crucial mediator of epithelialization, angiogenesis, and immune regulation [9]. ARG has been incorporated into various polymeric systems, including hydrogels and films, where it contributes to macrophage polarization, enhanced angiogenesis, and antimicrobial activity [17,18].

Royal jelly (RJ), a natural secretion from the hypopharyngeal and maxillary glands of bees, has antibacterial, anti-inflammatory, and antioxidant properties, hence facilitating tissue regeneration and wound healing [19]. RJ has also been investigated as a functional component in hydrogel-based dressings, demonstrating anti-inflammatory, antimicrobial, and wound healing-promoting effects [19,20].

Wound healing is a dynamic and precisely controlled process that advances through many different phases: hemostasis, inflammation, cellular proliferation, and tissue remodeling. The stages are regulated by intricate interactions among growth factors, enzymes, cytokines, ECM components, and host responses to microbial challenges [21]. The effective decrease in bacterial colonization and prompt resolution of inflammation are essential for accelerating the shift to the proliferative phase and facilitating granulation tissue development [22,23]. Conversely, severe or persistent inflammation might hinder healing by disturbing the equilibrium between collagen production and breakdown [24].

Preserving a hydrated wound environment with occlusive or semipermeable dressings is crucial for averting desiccation and crust development, as well as promoting re-epithelialization [25,26,27,28]. This process includes keratinocyte migration and proliferation, the formation of a provisional matrix, differentiation of the neoepidermis, and the repair of the dermo-epidermal junction [29,30,31,32,33,34,35]. Re-epithelialization, generally starting within 16–24 h post-injury, is governed by growth factors (EGF, HGF, FGFs, TGF, IGF-1, NGF, GM-CSF), integrins (αvβ5, αvβ6, α5β1), matrix metalloproteinases (MMPs), ECM components, and NO, which collectively modulate keratinocyte activation, migration, and keratin expression (K6, K16, K17) crucial for efficient wound closure [23,36,37,38,39,40,41].

The therapeutic management of re-epithelialization is dependent upon various factors, including wound type (e.g., burn, ulcer, acute, or chronic), depth (e.g., epidermal, superficial partial-thickness, deep partial-thickness, or full-thickness), surface area, patient comorbidities such as diabetes, susceptibility to infections, and the degree of exudate [21].

Tissue engineering methods using biomimetic scaffolds have garnered significant interest. Electrospun nanofibrous matrices (NFs), characterized by elevated surface-to-volume ratios, interconnected porosity, and ECM-like architecture, provide advantageous conditions for keratinocyte needs and migration, thereby facilitating rapid re-epithelization [22].

Nanofibrous dressings composed of natural or synthetic polymers, like collagen, hyaluronic acid (HA), chitosan (CH), alginate, or elastin, are typically biocompatible, biodegradable, and non-toxic [23,24,25].

The aim of this study was to biologically evaluate CS/PVA NFs functionalized with natural ACs, used either individually or in synergistic dual combinations (ARG, ALA, RJ, and CUR), in order to assess their multifunctional potential for wound healing and skin regeneration. The present study focuses on the biological evaluation of these systems using in vitro and in vivo assays, while the detailed physicochemical characterization of the nanofibrous scaffolds has been previously reported. Considerable focus was directed into clarifying the synergistic effects of co-loaded CS/PVA nanofibers on antioxidant, anti-inflammatory, and regenerative responses, using complementary in vitro experiments and an in vivo wound healing model.

## 2. Results

The physicochemical characteristics of CH/PVA-based electrospun NFs loaded with ACs were previously reported [42]. SEM analysis revealed the formation of uniform, bead-free nanofibers with a narrow diameter distribution, indicating stable electrospinning conditions. The obtained nanofibrous structures exhibited a high swelling capacity, which is particularly relevant for wound healing applications due to their ability to absorb exudate and maintain a moist microenvironment [42].

### 2.1. Antioxidant Assays

In inflammatory processes, reactive oxygen species (ROS) are produced, and excessive ROS generation surpasses the intrinsic antioxidant defense mechanism, resulting in oxidative stress. Antioxidant molecules are essential in rectifying this imbalance by neutralizing ROS and mitigating oxidative damage [37]. The integration of antioxidant elements into biomaterials constitutes an effective approach to alleviate oxidative stress at the wound site and facilitate the healing process.

**Figure 1:**
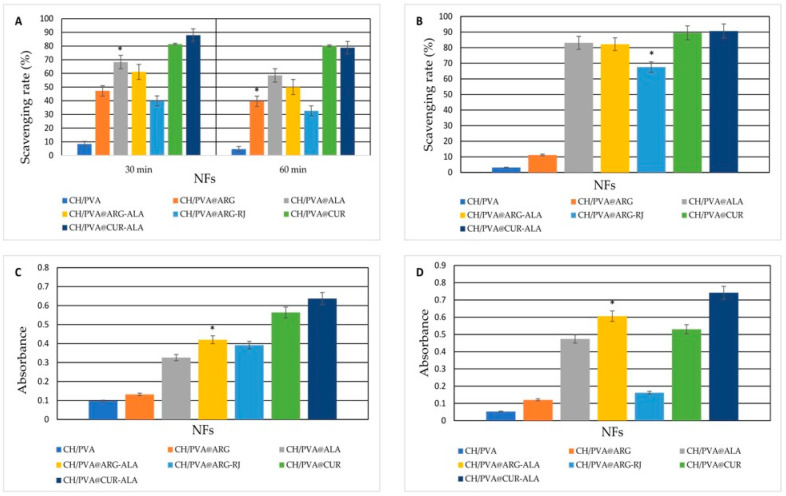
The antioxidant effects of CH/PVA@ACs: DPPH (**A**), ABTS (**B**), PRAP (**C**), and FRAP (**D**) * *p* ˂ 0.05 when compared to CH/PVA.

Figure 1A demonstrates that NFs containing CUR had significant DPPH radical scavenging activity. The CH/PVA@CUR NFs exhibited inhibition values of 81.47 ± 0.10% and 80.17 ± 0.44% after 30 and 60 min, respectively, but the co-loaded CH/PVA@CUR-ALA system shown superior activity at 30 min (87.91 ± 0.10%), suggesting a synergistic effect. The increased antiradical activity of CUR aligns with its antioxidant mechanism, which is based on tautomerism, as extensively documented in the literature [43]. Moreover, low quantities of ALA increased the scavenging action, consistent from other studies [1,37]. Accordingly, CH/PVA@ARG-ALA NFs exhibited higher DPPH inhibition (61.05 ± 0.62 after 30 min and 50.02 ± 0.65 after 60 min) than CH/PVA@ARG alone (47.21 ± 0.16 after 30 min and 39.60 ± 1.16 after 60 min). In contrast, the CH/PVA matrix without ACs showed only negligible antioxidant activity (8.48 ± 0.24% after 30 min and 4.63 ± 0.82% after 60 min), confirming the key contribution of the loaded ACs.

The ABTS assay (Figure 1B) further confirmed the strong antioxidant potential of the functionalized NFs. CH/PVA@ARG-RJ NFs showed a notable inhibition value (67.53 ± 0.87%), attributable to the major RJ protein (MRJP) fraction [40]. Furthermore, CUR- and ALA-based systems demonstrated superior ABTS scavenging activity, with the peak value seen for CH/PVA@CUR-ALA NFs (90.68 ± 0.21%). Consistent patterns were seen in the PRAP and FRAP experiments, whereby all AC-loaded NFs exhibited substantially elevated absorbance values compared to the CH/PVA control (0.09 ± 0.01 for PRAP and 0.05 ± 0.03 for FRAP). Referring to PRAP, very good results were obtained for CH/PVA@CUR-ALA NFs (0.63 ± 0.02), CH/PVA@CUR NFs (0.56 ± 0.05), CH/PVA@ARG-ALA NFs (0.42 ± 0.01), and CH/PVA@ARG-RJ NFs (0.39 ± 0.02). In the FRAP assay, the best antioxidant effect was observed for CH/PVA@CUR-ALA NFs (0.74 ± 0.05) and CH/PVA@ARG-ALA NFs (0.60 ± 0.05).

### 2.2. Protein Denaturation Assay

The protein denaturation assay was used to evaluate the anti-inflammatory efficacy of the produced nanofibrous systems, based on their capacity to protect proteins against heat aggregation, a phenomenon intimately linked to inflammatory responses. The inhibition of protein denaturation, which involves the breakdown of hydrogen, hydrophobic, and disulfide bonds, is regarded as a significant marker of anti-inflammatory efficacy [19]. Bovine serum albumin, a recognized model protein system [44,45] was used, and the experiment was conducted under physiological (PBS, pH 7.4) and acidic (AcBS, pH 5.5) conditions to replicate normal and inflamed wound environments.

**Figure 2:**
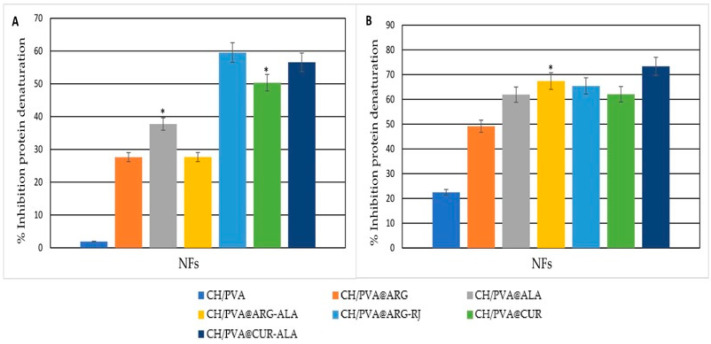
Inhibition of protein denaturation by CH/PVA@ACs NFs under PBS (**A**) and AcBS (**B**) conditions. * *p* < 0.05 when compared to CH/PVA.

CUR-loaded NFs had a notable protective effect against protein denaturation, with inhibition values of 50.33 ± 0.23% (CH/PVA@CUR) and 56.56 ± 2.27% (CH/PVA@CUR-ALA) at pH 7.4. In acidic conditions (pH 5.5), the CH/PVA@CUR system demonstrated increased inhibition (62.10 ± 0.11%), suggesting improved stability in an inflammatory-like environment (Figure 2).

ARG-containing NFs exhibited a more moderate protective effect, aligning with the literature findings that suggest that guanidinium groups preferentially engage in hydrogen bonding with polymers and co-loaded compounds, consequently restricting their interaction with proteins and their capacity to inhibit protein aggregation [46,47]. The inhibition values for CH/PVA@ARG NFs were 27.63 ± 0.21% at pH 7.4 and 49.17 ± 0.09% at pH 5.5. Conversely, ALA-containing systems exhibited enhanced protection, with inhibition values of 37.74 ± 0.67% (pH 7.4) and 61.95 ± 0.03% (pH 5.5), consistent with findings that demonstrate ALA’s greater stabilizing action against heat protein denaturation [48].

CH/PVA@ARG-RJ NFs exhibited a greater inhibition, with values of 59.53 ± 0.61% in PBS and 65.47 ± 0.03% in AcBS, respectively. This impact is mostly attributed to RJ, possibly due to its lipid constituents, including 10-hydroxy-2-decenoic acid and sebacic acid, recognized for their anti-inflammatory properties [49].

The results indicate that the inclusion of ACs, particularly CUR, ALA, and RJ, markedly improves the capacity of CH/PVA nanofibrous systems to protect proteins from denaturation in both physiological and inflammatory-mimicking environments, thereby endorsing their potential anti-inflammatory properties.

### 2.3. Hemolysis Assay

**Figure 3:**
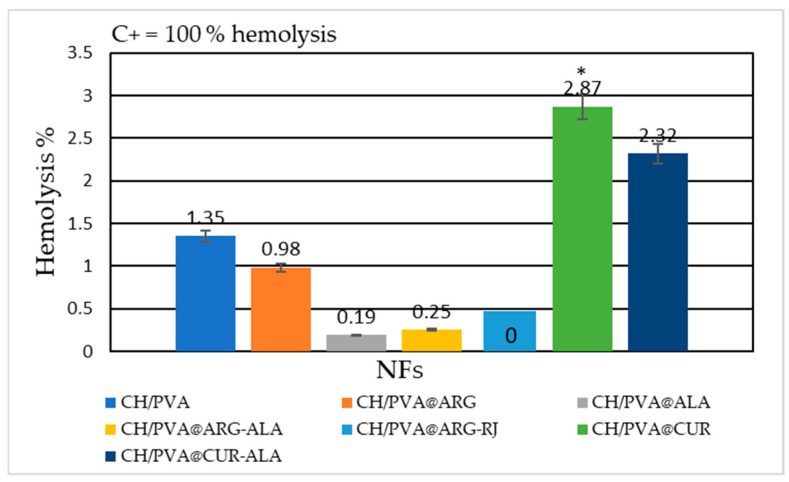
The hemolysis effect of CH/PVA@ACs NFs. * *p* ˂ 0.05 when compared to CH/PVA.

Assessment of hemocompatibility is a critical requirement for biomaterials intended for in vivo applications. Materials with a hemolysis rate under 5% are classified as non-hemolytic and appropriate for biomedical applications [35]. This study demonstrated that the CH/PVA@ARG-RJ NFs had 0% hemolysis, indicating exceptional hemocompatibility (Figure 3). The CH/PVA NFs exhibited a hemolysis value of 1.35 ± 0.18%, which was further decreased to 0.98 ± 0.31% for CH/PVA@ARG, 0.19 ± 0.10% for CH/PVA@ALA, and 0.25 ± 0.00% for CH/PVA@ARG-ALA. The CUR-containing systems, CH/PVA@CUR and CH/PVA@CUR-ALA, demonstrated somewhat elevated hemolysis values (2.87 ± 0.27% and 2.32 ± 0.27%, respectively); still, these values were far below the 5% criterion. Therefore, all of the developed nanofibrous systems are classed as hemocompatible, indicating their appropriateness for biomedical applications without the danger of acute hemolysis.

### 2.4. Cell Viability Assay

Although the intended application targets cutaneous wound healing, HGFs represent an appropriate cell model for evaluating the cytotoxicity of CH/PVA@ACs NFs, owing to their increased sensitivity to potentially toxic agents. This provides a more conservative and reliable assessment of biocompatibility compared with normal human dermal fibroblasts (NHDFs). No notable morphological or functional differences have been documented between the two fibroblast types [50]. 

**Figure 4:**
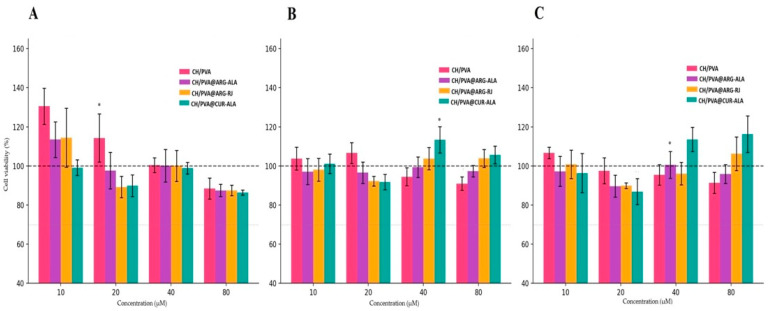
Cell viability (% of control) at three time points: 24 h (**A**), 48 h (**B**), and 72 h (**C**), * *p* < 0.001.

The effect of CH/PVA@ACs NFs on HGF viability was evaluated at three time points (24, 48, and 72 h) using four concentrations (10, 20, 40, and 80 µM). The unloaded CH/PVA scaffold was used to differentiate the inherent influence of the polymer matrix from the biological activity of the loaded ACs. One-way ANOVA demonstrated statistically significant differences across experimental groups at all assessed time points (24 h: F = 11.58, *p* < 0.0001; 48 h: F = 6.09, *p* < 0.0001; 72 h: F = 6.87, *p* < 0.0001) (Figure 4).

At 24 h, the CH/PVA scaffold significantly enhanced the cell viability at 10 µM (130.53 ± 9.27%) and 20 µM (114.33 ± 12.28%) in comparison to the control. The formulations CH/PVA@ARG-ALA and CH/PVA@ARG-RJ at 10 µM induced significant enhancements in viability (113.47 ± 9.09% and 114.41 ± 15.08%, respectively), however, these results were somewhat inferior than those recorded for the CH/PVA scaffold. CH/PVA@CUR-ALA did not differ significantly from the control at this time point.

At 48 h, the proliferative effect of the CH/PVA scaffold was no longer evident. Nonetheless, CH/PVA@CUR-ALA at 40 µM markedly enhanced the cell viability (113.40 ± 6.67%), although CH/PVA@ARG-ALA and CH/PVA@ARG-RJ exhibited similar results to the control.

At 72 h, CH/PVA@CUR-ALA had the most stable proliferative response, with significant increases in cell viability at 40 µM (113.54 ± 6.19%) and 80 µM (116.31 ± 9.32%). Conversely, the unloaded CH/PVA scaffold and the other formulations did not provide statistically significant effects at this time point.

Significantly, none of the evaluated formulations decreased the cell viability below the 70% threshold often used to signify cytotoxicity, thus affirming the general biocompatibility of both the scaffold and the loaded formulations. Among the CH/PVA@ACs, CH/PVA@CUR-ALA exhibited the most significant and enduring proliferative effect, especially at doses of 40 and 80 µM after extended exposure.

### 2.5. Wound Healing

#### 2.5.1. Macroscopic Analysis

The in vivo wound contraction data (Table 1) demonstrate that all CH/PVA@ACs significantly accelerated wound closure compared to the negative control (Medicomp^®^ PAUL HARTMANN AG, Heidenheim an der Brenz, Germany) (*p* < 0.05 at all evaluated time points). A faster reduction in wound area was already evident from day 6, when the experimental groups exhibited markedly higher contraction percentages (40.18 ± 1.81% for CH/PVA@ARG-RJ; 46.05 ± 2.05% for CH/PVA@ARG-ALA) than the Medicomp^®^ group (32.40 ± 2.05%).

Among the tested formulations, CH/PVA@ARG-ALA and CH/PVA@ARG-RJ showed the most rapid healing effect, reaching over 95% wound contraction by day 12 and nearly complete closure (>99%) by day 18, outperforming both the CH/PVA and the commercial positive control (Exufiber^®^ Mölnlycke Health Care, Göteborg, Sweden). The CH/PVA@CUR-ALA group also significantly improved wound closure compared to the controls (Exufiber^®^, Medicomp^®^), although with a slightly slower progression, reaching 96.98 ± 0.15% by day 18.

The CH/PVA matrix alone promoted wound healing compared to the negative control; however, its performance remained inferior to that of the AC-loaded systems, particularly at early and intermediate time points (days 3–12). Notably, the commercial positive control (Exufiber^®^) showed slower wound contraction than the best-performing experimental groups, especially at days 6 and 9, confirming the superior efficacy of the newly developed nanofibrous dressings.

**Figure 5:**
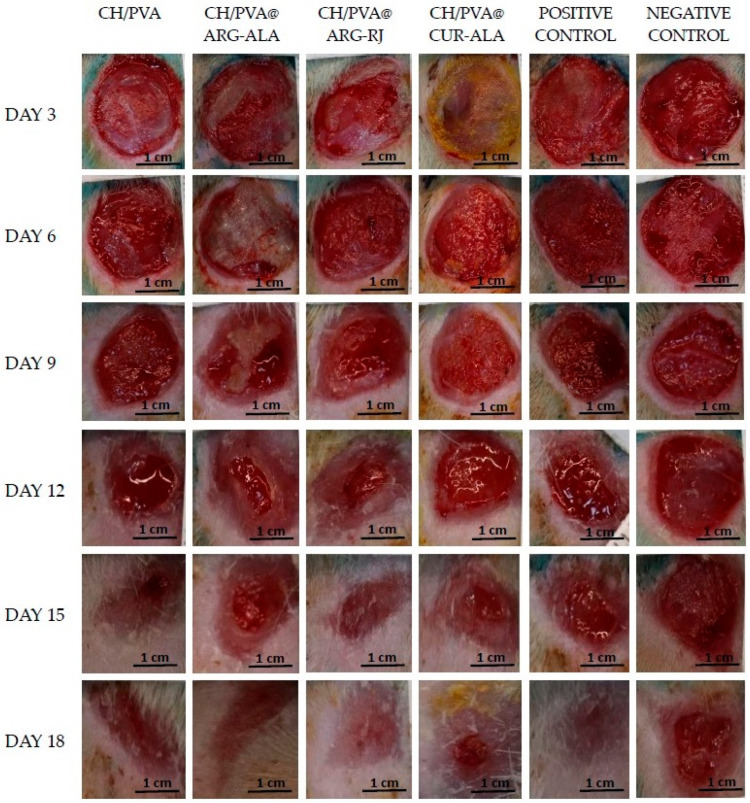
Sequential macroscopic evaluation of excisional wound healing at different time points following treatment with CH/PVA@ACs NFs.

At all evaluated time points, the experimental groups exhibited a reduced wound area, decreased inflammatory signs, and less perilesional erythema compared to the negative control (Medicomp^®^). In addition, hair regrowth was observed in the treated wound areas by day 15 (Figure 5). No signs of allergic reactions, tissue rejection, purulent discharge, or infection were observed throughout the study period, indicating the good in vivo biocompatibility of the tested materials. Moreover, the CH/PVA@ACs nanofibrous systems exhibited wound healing effects comparable to the positive control (Exufiber^®^), particularly from day 9 onward.

#### 2.5.2. Histopathological Analysis

On day 6, the wounds were partially closed and covered by a crust composed of coagulated blood, fibrin, infiltrating polymorphonuclear cells (PMNs), and necrotic tissue debris. Crust formation was observed at the wound periphery in the CH/PVA, CH/PVA@ARG-RJ, CH/PVA@ALA-CUR, and Exufiber^®^ groups, with thicker crusts covering approximately 40% of the wound surface in all groups. Histological analysis revealed a well-formed, vascularized granulation tissue abundant in mononuclear inflammatory cells, including macrophages, plasma cells, lymphocytes, and fibroblasts.

**Figure 6:**
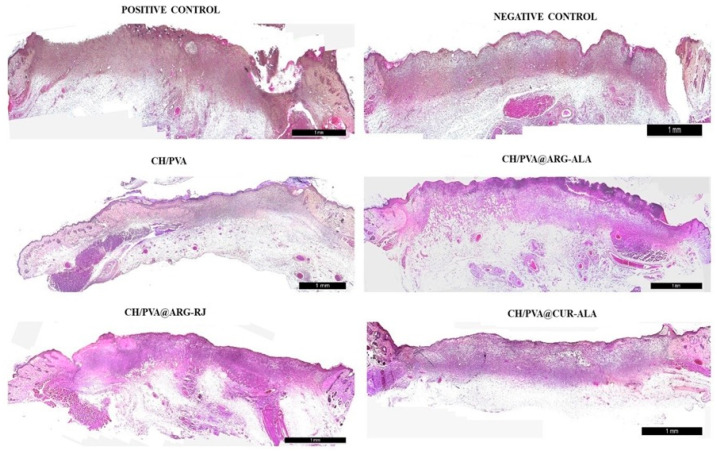
Histological changes in excisional wounds treated with CH/PVA@ACs NFs (day 6) (H&E, scale bar 1 mm).

A significant concentration of newly created blood vessels was seen throughout the wound area, with granulation tissue being more pronounced in the subepidermal region compared to the deeper dermis (Figure 6).

**Figure 7:**
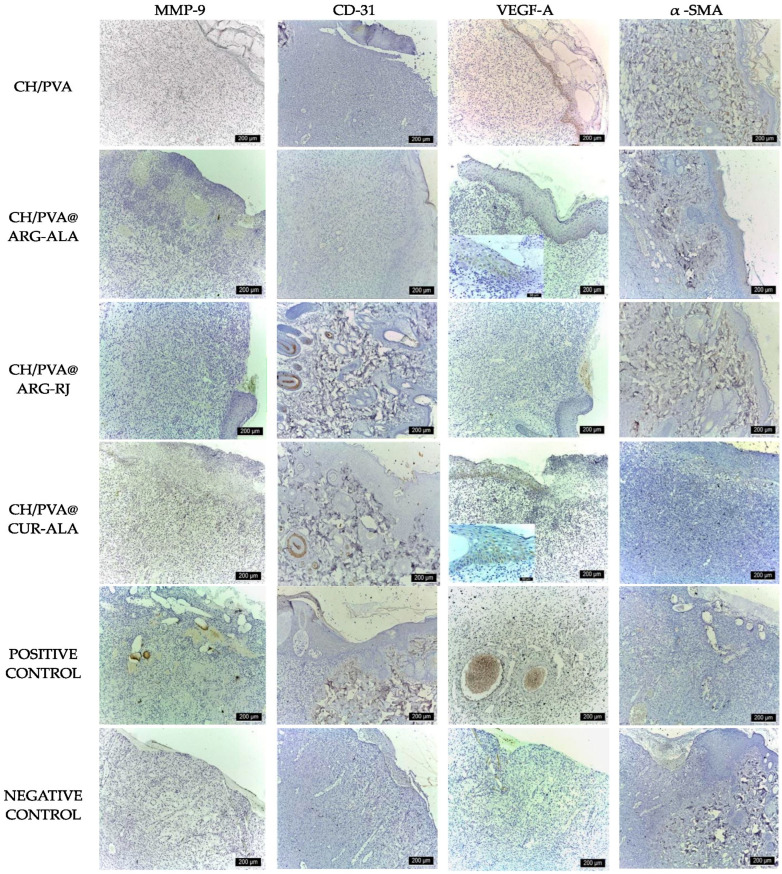
Immunohistochemical expression of wound healing markers in excisional wounds treated with CH/PVA@ACs NFs (day 6) (scale bar 200 µm).

Immunohistochemical analysis of tissue samples collected on day 6 corroborated these findings, as all experimental groups demonstrated the strong expression of critical wound-healing markers. MMP-9 expression indicated active ECM remodeling, while CD31 positivity identified endothelial cells and newly formed vascular structures. VEGF-A was mostly expressed in the basal layer of the epidermis and in the neocapillaries, indicating active angiogenesis. In addition, α-SMA expression in vascular smooth muscle cells highlighted ongoing vascular maturation and tissue remodeling processes (Figure 7).

**Figure 8:**
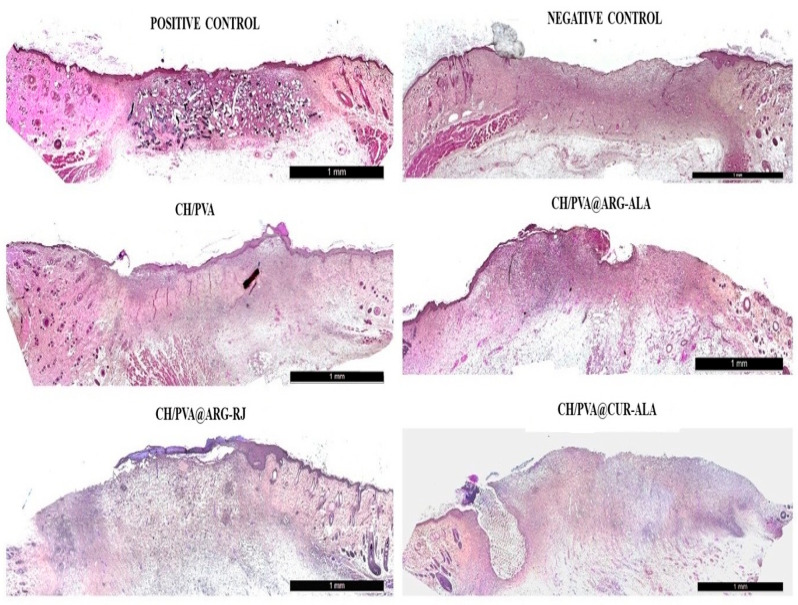
Histological changes in excisional wounds treated with CH/PVA@ACs NFs (day 12) (H&E, scale bar 1 mm).

On day 12, corresponding to the early proliferative phase, the experimental groups exhibited more advanced wound healing than the negative control (Medicomp^®^). A continuous keratinocyte bridge covered approximately 70–80% of the lesion area in the treated groups compared to only about 30% in the Medicomp^®^ group. Histological analysis demonstrated well-formed fibrovascular granulation tissue, marked by an abundance of migrating cells, thin and disordered collagen fibers, and a sparse presence of newly created blood vessels (Figure 8).

**Figure 9:**
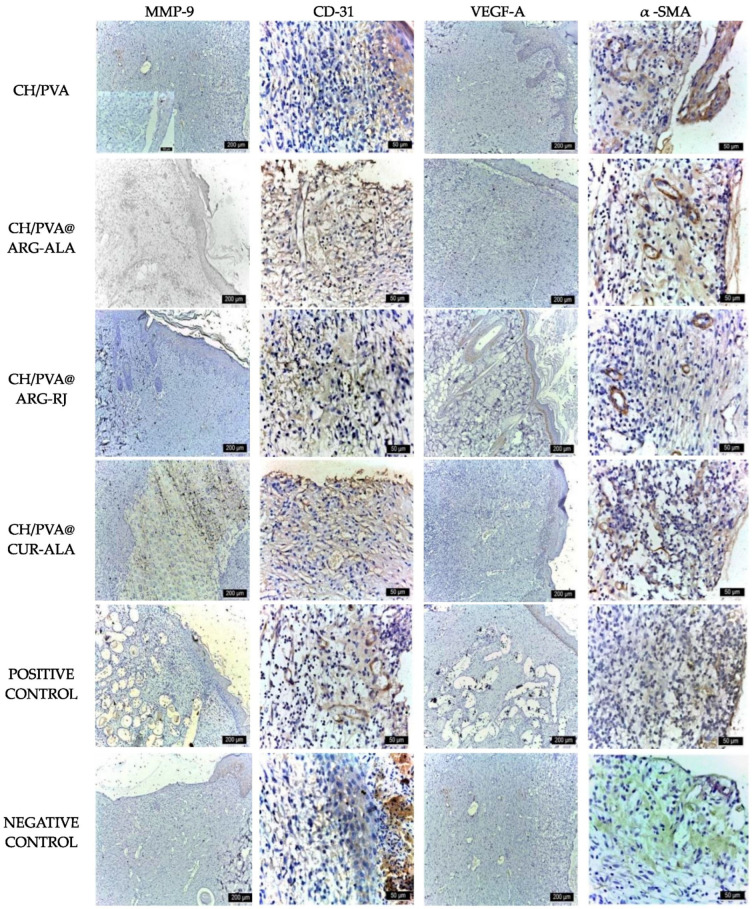
Immunohistochemical expression of wound healing markers in excisional wounds treated with CH/PVA@ACs NFs (day 12) (scale bar 200 µm for MMP9, VEGF-A and 50 µm for CD31 and α-SMA).

The immunohistochemical analysis performed on day 12 corroborated these results. All experimental groups, with the exception of the Medicomp^®^ group, exhibited strong expression of critical wound-healing markers. CD31 mostly marked endothelial cells of nascent capillaries and macrophages, while α-SMA delineated vascular smooth muscle cells and myofibroblasts. VEGF-A was mostly expressed in the basal layer of the epidermis and in neocapillaries, indicating active angiogenesis. Conversely, MMP-9 expression was higher in the untreated control, indicating enhanced extracellular matrix breakdown and decreased tissue rebuilding efficacy in the absence of biomaterials (Figure 9).

**Figure 10:**
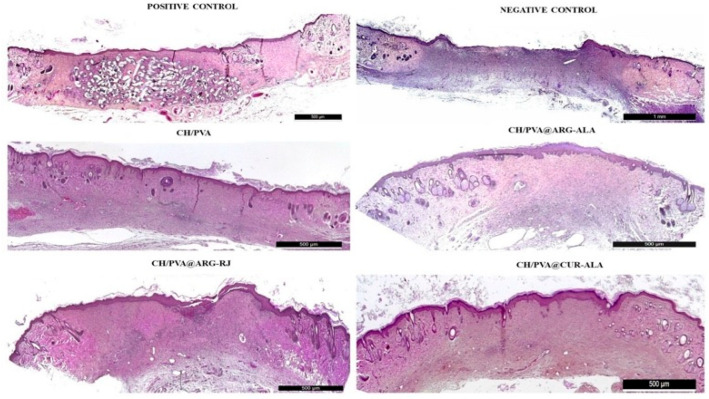
Histological changes in excisional wounds treated with CH/PVA@ACs NFs (day 18) (H&E, scale bars: 500 µm—samples and positive control and 1 mm—negative control).

On day 18, the experimental groups demonstrated enhanced wound healing, characterized by a completely re-epithelialized and well-structured epidermis with a robust keratin layer, alongside a dermis comprising organized connective tissue and collagen fibers, with only sparsely scattered blood vessels. In addition, the presence of hair follicles and sebaceous glands indicated advanced tissue remodeling. In contrast, the untreated control (Medicomp^®^ group) showed an uneven epidermis, a thin keratin layer, and persistent inflammatory cell infiltrates (Figure 10).

**Figure 11:**
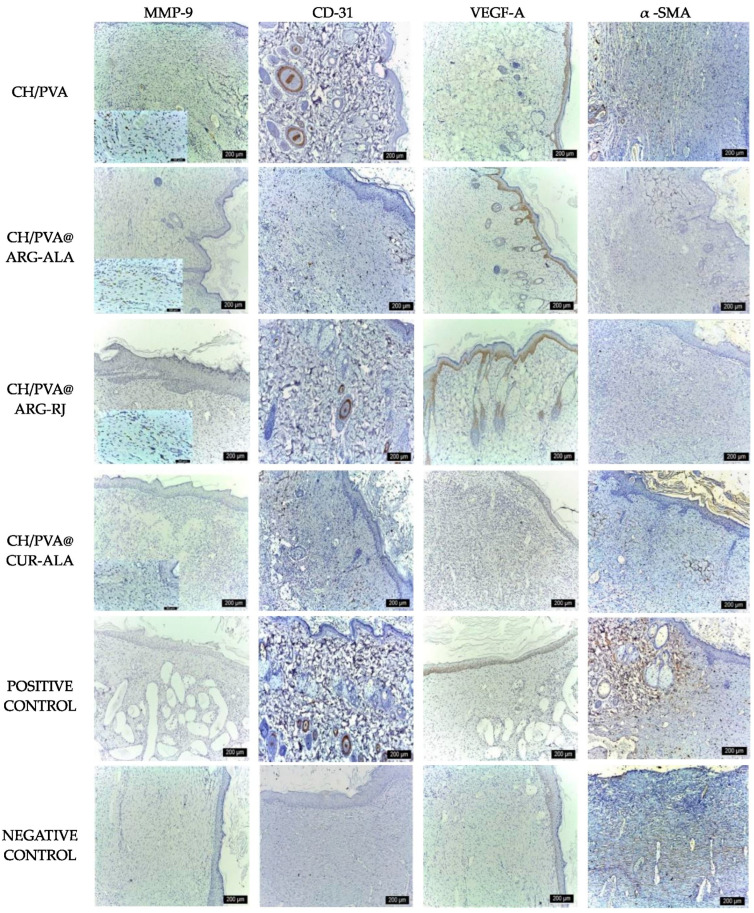
Immunohistochemical expression of wound healing markers in excisional wounds treated with CH/PVA@ACs NFs (day 18) (scale bar 200 µm).

Immunohistochemical analysis at day 18 revealed variable expression patterns of the investigated markers. CD31 mainly labels endothelial cells in residual neocapillaries and occasional macrophages. α-SMA marked vascular smooth muscle cells and myofibroblasts, with more pronounced staining seen in the Medicomp^®^ group, indicating delayed remodeling. VEGF-A expression was seen in the basal layer of the epidermis and in the epithelial sheaths of hair follicles, indicative of active tissue development. MMP-9 exhibited moderate expression in the Medicomp^®^ group, while lower levels were seen in the treatment groups, mostly concentrated in the deep dermis, suggesting less extracellular matrix destruction and enhanced tissue remodeling in the experimental groups (Figure 11).

In conclusion, the current findings indicate that CH/PVA NFs functionalized with ACs (CH/PVA@ARG-ALA, CH/PVA@ARG-RJ, CH/PVA@CUR-ALA) display greater effectiveness in wound healing, facilitating accelerated tissue remodeling, improved angiogenesis, and superior tissue remodeling. The therapeutic results obtained with the developed nanofibrous systems were similar to those seen with the positive control (Exufiber^®^). These results validate the superior in vivo biocompatibility and significant therapeutic potential of the developed biomaterials for skin regeneration applications.

## 3. Discussion

The previously reported physicochemical properties [42] confirm that the CH/PVA@ACs nanofibrous systems provide a suitable structural platform for biomedical applications. The uniform morphology and controlled fiber diameter indicate a stable jet formation and optimized solution properties, which are essential for reproducible scaffold fabrication. Moreover, the swelling capacity supports its potential to efficiently manage wound exudate, thereby promoting a favorable environment for tissue regeneration. These characteristics are critical for ensuring both the structural integrity and functional performance of the nanofibrous scaffolds in wound healing applications.

The selection of AC concentrations was based on a combined approach involving literature-reported effective ranges and experimental optimization. Previous studies have demonstrated the therapeutic potential of ARG, ALA, CUR, and RJ in wound healing applications [14,15,18,51]. However, beyond their biological activity, their influence on the physicochemical properties of the polymeric solution, such as viscosity and electrical conductivity, were carefully considered, as these parameters directly affect jet stability and fiber formation during electrospinning. Therefore, the selected concentrations represent an optimal balance between therapeutic efficacy, process stability, and the preservation of uniform nanofibrous morphology.

The incorporation of ACs (ARG, ALA, RJ, CUR) into the CH/PVA nanofibrous matrix was previously confirmed by FTIR analysis, indicating the presence of intermolecular interactions that contribute to system stability. Furthermore, the release behavior of the ACs has been shown to be pH-dependent, which is particularly relevant in the context of wound healing, where local pH variations occur during different stages of tissue repair [52]. Under mildly acidic conditions (pH 5.5), the release is accelerated and involves both diffusion-controlled and anomalous transport mechanisms, while at physiological pH (pH 7.4), diffusion remains the dominant mechanism. Importantly, the simultaneous incorporation of multiple ACs did not adversely affect their individual release profiles, suggesting good compatibility within the polymeric matrix and supporting the feasibility of multi-component therapeutic systems.

The in vitro assessment of antioxidant potential indicates that the integration of ACs into the CH/PVA polymeric matrix substantially improves the radical scavenging ability of the nanofibrous systems. These findings align with previous publications detailing the synergistic function of ARG, ALA, RJ, and CUR in regulating oxidative stress at the wound site [23,43,46,53]. The CH/PVA@CUR-ALA formulation demonstrated the highest antioxidant efficacy (90.68 ± 0.21% ABTS inhibition), highlighting the essential role of phenolic compounds and bioactive amino acids in mitigating the overproduction of ROS during the inflammatory stage of wound healing [54,55]. The improved antioxidant efficacy was also validated by the protein denaturation inhibition experiment, where CH/PVA@CUR-ALA NFs had the most significant inhibitory effect (62.10 ± 0.11% at pH 5.5). This outcome reinforces the intricate relationship between oxidative stress modulation and the inhibition of the inflammatory cascade, as previously emphasized for CUR-loaded CH nanoparticles [56] and multifunctional CH/PVA/gelatin hydrogels [57], which demonstrated a reduction in TNF-α levels and an enhancement of collagen deposition.

Although in vitro antioxidant assays are widely used for the rapid screening of antioxidant activity, it is important to acknowledge their inherent limitations. These methods are based on simplified in vitro chemical reactions, primarily involving electron transfer mechanisms, and employ synthetic radicals that do not fully replicate the complex oxidative processes occurring in biological systems. Therefore, the results obtained in this study should be interpreted as indicative of the intrinsic antioxidant potential of the developed nanofibrous systems, serving as a preliminary evaluation that complements future biological investigations.

The anti-inflammatory activity assessed using the BSA protein denaturation assay represents a widely accepted in vitro method for the preliminary screening of compounds with potential anti-inflammatory properties. This assay is based on the inhibition of protein denaturation, a process associated with inflammatory conditions and loss of protein function. However, it should be noted that this method provides only indirect evidence of anti-inflammatory activity and does not fully capture the complexity of inflammatory responses at the cellular and molecular levels, such as cytokine production, NO release, or immune cell activation.

Hemocompatibility testing indicated hemolysis values between 0% (CH/PVA@ARG-RJ) and 2.87% (CH/PVA@CUR), both of which are well below the clinically recognized threshold of 5% for safety. The findings align with those documented for analogous CH/PVA-based systems, which demonstrated hemolysis rates between 0.08% and 3.19% [51], thereby affirming the appropriateness of the proposed biomaterials for applications requiring direct blood contact.

The use of HGFs as a cellular model is supported by their well-documented similarities with dermal fibroblasts, particularly in terms of gene expression profiles related to extracellular matrix organization, cell proliferation, and tissue remodeling [50]. Moreover, HGFs are characterized by a higher proliferative capacity and an enhanced regenerative phenotype, often associated with reduced scar formation, which makes them a sensitive model for evaluating biomaterials intended for tissue repair. Although HGFs are not skin-derived cells, their biological behavior and involvement in wound healing processes make them a relevant model for preliminary cytocompatibility assessment. The cell viability assay demonstrated that there were no cytotoxic effects under any tested conditions, thereby affirming the excellent biocompatibility of both the unloaded scaffold (CH/PVA) and the loaded formulations (CH/PVA@ACs), consistent with prior reports detailing the favorable safety profile of CH/PVA nanofibers [58].

The temporary proliferative response seen for CH/PVA@ARG-ALA and CH/PVA@ARG-RJ at early time points (24 h, 10 µM) seems to be mostly linked to the scaffold rather than the embedded ACs, since similar effects were also shown for the unloaded CH/PVA at similar concentrations. Such scaffold-mediated stimulation has been previously reported for fibrous biomaterials and is commonly attributed to enhanced cell–matrix interactions that promote early cell adhesion and metabolic activation rather than AC-specific proliferative signaling. In contrast, CH/PVA@CUR-ALA exhibited a distinct, time- and concentration-dependent proliferative profile, which persisted and intensified up to 72 h and was not observed for the unloaded scaffold. This behavior suggests a potential contribution of the incorporated ACs to the observed proliferative response. Collectively, these results suggest that CH/PVA@CUR-ALA is the most promising formulation, especially for applications necessitating improved cellular proliferation, such as wound healing.

The Wistar rat excisional wound model is widely used in wound healing research due to its reproducibility and its ability to simulate the main physiological stages of tissue repair observed in humans. This model enables the in vivo assessment of the regenerative potential and therapeutic efficacy of nanofibrous wound dressings under controlled conditions.

All experimental groups demonstrated superior wound contraction relative to the CH/PVA scaffold, with the greatest significant effects seen for CH/PVA@ARG-ALA NFs (99.91 ± 0.168) and CH/PVA@ARG-RJ (99.94 ± 0.59) on day 18. A significant effect was also seen for CH/PVA@CUR-ALA (96.98 ± 0.15%). At the end of the experiment (day 18), all evaluated formulations demonstrated wound contraction values comparable to the positive control, Exufiber^®^ (95.06 ± 0.12%). These results align with other studies indicating that CH/PVA-based nanofibrous dressings embedded with ACs markedly enhance wound closure by promoting cell migration and fibroblast proliferation [55,58]. Moreover, the histological identification of hair follicles and sebaceous glands at day 18, along with a fully organized epidermis and well-structured connective tissue, not only signifies accelerated wound closure but also the restoration of normal skin architecture and function, consistent with the recent literature [59,60]. In addition, no allergic responses, rejection symptoms, purulent discharge, or infections were detected over the 18-day testing period.

When evaluated in conjunction with the hemocompatibility and cytotoxicity data, these findings confirm the superior in vivo biocompatibility of the engineered nanofibrous systems, consistent with prior research affirming the safety of CH/PVA-based NFs for wound healing applications [61].

## 4. Materials and Methods

### 4.1. Materials

Chitosan (CH; medium molecular weight, degree of deacetylation 75–85%), L-arginine (ARG; ≥98%, mp 226–230 °C), allantoin (ALA; ≥98%, mp 230 °C), 2,2-diphenyl-1-picrylhydrazyl (DPPH), 2,2′-azino-bis(3-ethylbenzothiazoline-6-sulfonic acid) (ABTS), glacial acetic acid (≥99.7%), sodium bicarbonate (NaHCO_3_), sodium chloride (NaCl), monosodium phosphate (NaH_2_PO_4_), potassium persulfate (K_2_S_2_O_8_), potassium ferricyanide (K_3_[Fe(CN)_6_]), ferric chloride (FeCl_3_), ammonium molybdate ((NH_4_)_2_MoO_4_), sulfuric acid (H_2_SO_4_), disodium hydrogen phosphate (Na_2_HPO_4_), and trichloroacetic acid were purchased from Sigma-Aldrich (Merck, Romania). Poly(vinyl alcohol) (PVA; 87–89% hydrolyzed, high molecular weight, average M.W. 88.000–97.000 Da) was obtained from Alfa Aesar (Alfa Aesar, Ward Hill, MA, USA). Curcumin (CUR; analytical standard, M.W. 368.38 g/mol), distilled water, and deionized water were obtained from Supelco (Bellefonte, PA, USA). Pure royal jelly (RJ), containing 1.59% (*w*/*w*) 10-hydroxy-2-decenoic acid, was supplied by Melidava (Bucharest, Romania). Absolute ethanol (99.5%) was purchased from Chimreactiv (Bucharest, Romania).

Human gingival fibroblasts (HGFs) were obtained from Cytion GmbH (Berlin, Germany). α-Minimum Essential Medium (α-MEM) was purchased from Biowest (Nuaillé, France). Dulbecco’s phosphate-buffered saline (DPBS), fetal bovine serum (FBS), and penicillin–streptomycin (1%) were purchased from PAN-Biotech GmbH (Aidenbach, Germany). Trypsin–EDTA (0.25%) was obtained from Lonza (Basel, Switzerland), and thiazolyl blue tetrazolium bromide (MTT) from Biosynth (Staad, Switzerland).

Isoflurane was purchased from Wellona Pharma (Surat, India), and tramadol (50 mg/mL) from Farmexim (Bucharest, Romania). Citrate buffer (10 mM, pH 6) was obtained from Novocastra(Leica Biosystems, Newcastle upon Tyne, UK). Hydrogen peroxide (H_2_O_2_, 3%), 3,3′-diaminobenzidine (DAB), and hematoxylin were obtained from Novolink^TM^ Polymer Detection Systems (Leica Biosystems, Newcastle upon Tyne, UK).

Primary antibodies included anti-MMP-9 (C-20; sc-6840), anti-CD31 (ER-MP12; MA1-40074), anti-VEGFA (VG1; MA1-16629), and anti-α-smooth muscle actin (α-SMA; clone 1A4; ab7817). Superfrost™ microscope slides were purchased from Laboratorium (Bucharest, Romania).

CH/PVA nanofibrous scaffolds (CH/PVA NFs) and CH/PVA nanofibrous scaffolds loaded with natural bioactive compounds (CH/PVA@ACs: CH/PVA@ALA, CH/PVA@ARG-ALA, and CH/PVA@CUR-ALA) were prepared in our laboratory.

Wistar rats were obtained from the Cantacuzino Institute (Bucharest, Romania). Exufiber^®^ was purchased from Mölnlycke Health Care (Göteborg, Sweden), sterile gauze (Medicomp^®^) from PAUL HARTMANN (Heidenheim an der Brenz, Germany).

### 4.2. Methods

#### 4.2.1. Preparation of CH/PVA@ACs NFs

The preparation of CH/PVA@ACs NFs was previously reported by our research group [42]. Briefly, the nanofibrous scaffolds were fabricated by electrospinning of CH/PVA@ACs polymeric solutions prepared at a fixed CH:PVA volume ratio of 1:3 loaded with ACs including ARG (3%, *w*/*v*), ALA (3%, 4%, *w*/*v*), CUR (3%, *w*/*v*), and RJ (3%, equivalent to 50 mg of 10-HAD), either as a single AC (ARG, ALA, CUR) or in synergistic dual combinations (ARG-ALA, ARG-RJ, and ALA-CUR). The concentrations of ACs were selected based on the literature data and preliminary experimental optimization, ensuring both biological relevance and compatibility with the electrospinning process. Depending on the formulation, the electrospinning flow rate ranged from 0.3 to 0.4 mL/h, the applied voltage from 15 to 19 kV, and the tip-to-collector distance from 16 to 27 cm. These parameters were individually optimized to obtain uniform, continuous, and bead-free NFs [42]. Prior to performing the hemolysis assay and the in vivo experiments, the CH/PVA@ACs NFs samples were sterilized by UV-C irradiation for 30 min (15 min on each side) at a distance of approximately 15 cm. No additional pre-conditioning step (e.g., incubation in PBS or culture media) was performed before biological testing. This approach was adopted to preserve the structural integrity of the nanofibers and to prevent premature release of the incorporated ACs [62].

#### 4.2.2. In Vitro Biological Assays

##### Antioxidant Assays

The radical scavenging activity of CH/PVA@ACs NFs against DPPH and ABTS, as well as the total antioxidant capacity and ferric reducing antioxidant power, was evaluated.

##### DPPH Radical Scavenging Assay

The DPPH radical scavenging activity was assessed using the technique described by Solaberrieta et al. [28] with some changes. A 0.1 mM DPPH solution was prepared in absolute ethanol, and its absorbance was calibrated at 1.0 at 517 nm. Subsequently, 2 mL of freshly prepared DPPH solution was added to 10 mg of CH/PVA@ACs NFs, corresponding to an Ns concentration of 5 mg/mL. The samples were incubated for 30 min and 60 min at room temperature in the dark. After incubation, 250 µL of each sample supernatant was transferred to a 96-well plate, and the absorbance was measured at 517 nm using a microplate reader (FluoSTAR Omega BMG Labtech, Ortenberg, Germany). A negative control, containing DPPH solution and ethanol (150 µL DPPH and 100 µL ethanol) was used. All measurements were performed in triplicate, and the results were expressed as the mean ± standard deviation (SD). The DPPH radical scavenging activity was calculated as the percentage inhibition (I%) using the following formula [28]:

Inhibition (%) = (A_control_ − A_sample_)/A_control_ × 100 (1)

where A_control_ represents the absorbance of DPPH solution after 30 min and 60 min, respectively, and A_sample_ represents the absorbance of the samples at the corresponding time points.

##### ABTS^+^• Radical Scavenging Assay

The ABTS radical scavenging activity was evaluated according to the method reported by Quispe et al. [30] with minor modifications. The ABTS radical cation (ABTS^+^•) was generated by mixing a 7 mM ABTS solution with a 2.45 mM K_2_S_2_O_8_ solution in a 1:1 (*v*/*v*) ratio and allowing the reaction to proceed in the dark for 24 h. Prior to analysis, the ABTS^+^• solution was diluted with absolute ethanol to obtain an absorbance of 0.7 ± 0.2 at 734 nm. Subsequently, 2 mL of the freshly prepared ABTS^+^• solution was added to 10 mg of the CH/PVA@ACs NF samples. After incubation under gentle shaking, 250 µL of each sample supernatant was transferred to a 96-well plate, and the absorbance was measured at 734 nm after six min using a microplate reader (FluoSTAR Omega BMG Labtech). A negative control consisting of 150 µL ABTS solution mixed with 100 µL ethanol was used. All measurements were performed in triplicate, and the results were expressed as the mean ± SD. The ABTS radical scavenging activity was calculated as the percentage inhibition (I%) using the following formula [30]:

ABTS scavenging activity (%) = A_control_ − A_sample_/A_control_ × 100 (2)

where A_control_ represents the absorbance of the ABTS solution and A_sample_ represents the absorbance of the sample.

##### Ferric Reducing Antioxidant Power (FRAP) Assay

The method involves the reduction of ferricyanide to ferrocyanide in the presence of electron-donating chemicals, leading to the formation of a blue complex that can be measured spectrophotometrically at 700 nm [31]. In summary, 10 mg of CH/PVA@ACs NF samples were combined with 0.5 mL of phosphate buffer solution (PBS, pH 6.6) and 0.5 mL of freshly prepared K_3_[Fe(CN)_6_] solution (1% *w*/*v*). The mixtures were incubated at 50 °C for 20 min, after which 0.5 mL of 10% (*w*/*v*) trichloroacetic acid was added. Subsequently, 20 µL of each sample supernatant was transferred in triplicate to a 96-well plate, to which 180 µL of distilled water and 50 µL of 0.1% *w*/*v* FeCl_3_ solution were added. After 10 min of incubation at room temperature, the absorbance was measured at 700 nm against a reagent blank using a microplate reader (FluoSTAR Omega BMG Labtech) [32]. Higher absorbance values indicate greater antioxidant activity. All measurements were performed in triplicate, and the results are expressed as the mean ± SD. An increase in optical density indicates a higher antioxidant activity.

##### Phosphomolybdenum Reducing Antioxidant Power (PRAP) Assay

The total antioxidant capacity was evaluated using the phosphomolybdenum reducing antioxidant power (PRAP) assay, a quantitative spectrophotometric method based on the reduction of Mo(VI) to Mo(V) in an acidic environment, resulting in the formation of a blue-green phosphomolybdenum complex measurable at 695 nm. Briefly, 2 mL of reagent solution, consisting of 4 mM (NH_4_)_2_MoO_4_, 0.6 M H_2_SO_4_, and 28 mM Na_2_HPO_4_ prepared in bidistilled water, were added to 10 mg of CH/PVA@ACs NF samples. The mixtures were incubated at 95 °C for 90 min and subsequently cooled to room temperature. The absorbance was measured at 695 nm against a reagent blank using a microplate reader (FluoSTAR Omega BMG Labtech) [32]. Higher absorbance values indicate greater antioxidant activity. The measurements were performed in triplicate, and the results are expressed as the mean ± SD.

##### Protein Denaturation Assay

The in vitro anti-inflammatory effects of the AC-loaded CH/PVA NFs were evaluated using the protein denaturation inhibition assay [44,45] with minor modifications. A total of 10 mg of CH/PVA@ACs NF samples were dissolved in PBS (pH 7.4) or acetate buffer solution (AcBS, pH 5.5) depending on the desired pH until a final volume of 2 mL was obtained. As a control for the albumin denaturation process, 2 mL of buffer solutions (PBS/AcBS) were used. Subsequently, 0.2 mL of bovine serum albumin solution (1% *w*/*v*) was added to the 2 mL of buffer solution and thoroughly mixed. The 10 mg CH/PVA@ACs NF samples were incubated for 15 min at 37 °C and then heated for 2 min at 80 °C. After cooling, spectrophotometric turbidimetry was measured using a multimode microplate reader (FluoSTAR Omega BMG Labtech) at 660 nm, and the inhibition percentage (I%) was calculated according to the following formula [45]:

Inhibition (%) = A_control_ − A_sample_/A_control_ × 100 (3)

where A_control_ and A_sample_ represent the absorbance value of the control and the samples.

##### Hemolysis Assay

As wound-healing materials may come into direct contact with blood, their hemocompatibility was evaluated by a hemolysis assay. The method described by Das et al. [35] was used with minor modifications. Fresh human blood was collected from healthy volunteers into heparinized tubes and stored at 4 °C prior to use. The blood was diluted with 0.9% (*w*/*v*) NaCl at a ratio of 4:5 (*v*/*v*). The CH/PVA@ACs NF samples were cut into 1 × 1 cm^2^ specimens and immersed in 2 mL of 0.9% NaCl, followed by incubation for 30 min at 37 °C. A positive control (pC), corresponding to 100% hemolysis, consisted of 2 mL distilled water, and a negative control (nC), corresponding to 0% hemolysis, consisted of 2 mL 0.9% NaCl, were also used. Subsequently, 50 µL of diluted blood was added to each sample and control, and the mixtures were incubated for 1 h at 37 °C. After incubation, the samples were centrifuged at 1500 rpm for 10 min. Aliquots (250 µL) of the samples/controls were collected in triplicate, and the absorbance was measured at 545 nm using a microplate reader (FluoSTAR Omega BMG Labtech). The percentage of hemolysis value was calculated using the following formula [35]:

Hemolysis (%) = (A_s_ − A_nC_)/(A_pC_ − A_nC_) × 100 (4)

where A_s_ is the absorbance of the sample, A_nC_ is the absorbance of the negative control, and A_pC_ is the absorbance of the positive control. The experiments were performed in triplicate, and the results are expressed as the mean ± SD.

##### Cell Viability Assay

HGF was cultured in α-MEM, supplemented with 10% FBS and 1% penicillin–streptomycin at 37 °C in a humidified atmosphere containing 5% CO_2_. Cells were seeded at a density of 5000 cells/well in 96-well plates and incubated for 24 h to allow cell adhesion. Following incubation, cells were treated with selected CH/PVA@ACs polymeric solution samples at different concentrations (80, 40, 20 and 10 µM). After 24, 48, and 72 h of treatment, MTT solution (prepared by dissolving MTT in sterile PBS and subsequently diluting in pre-warmed serum-free culture medium) was added to each well to achieve a final concentration of 0.5 mg/mL. The plates were incubated at 37 °C for 2–3 h to allow viable cells to reduce MTT to insoluble formazan crystals. Subsequently, the medium was carefully removed, and the formed formazan crystals were dissolved in 100 µL DMSO per well. The plates were shaken for 5–10 min to ensure complete solubilization. Absorbance was measured at 570 nm using a microplate reader (EZ Read 400, Microplate Reader, Biochrom, UK). Cell viability (%) was calculated by normalizing the absorbance of treated samples to that of the untreated control cells, which were considered 100% viable [63].

#### 4.2.3. In Vivo Biological Assay

##### Animals

Adult male Wistar rats weighing 300–400 g were used. The animals were obtained from an accredited laboratory animal facility and were clinically healthy at the beginning of the experiment. They were housed in individually ventilated cages (IVCs) inside the animal facility at the Advanced Research and Development Center for Experimental Medicine, “Prof. Ostin C. Mungiu” (CEMEX). The animals were kept under conventional husbandry settings, which included a controlled room temperature (20 ± 4 °C), relative humidity (50 ± 5%), and a regulated light–dark cycle, with ad libitum access to water and standard chow.

Following acclimation, animals were weighed, labeled, and randomly allocated into six experimental groups (n = 6 per group). The sample size was selected in accordance with the 3R principles (Replacement, Reduction, Refinement) and based on previously reported wound healing models employing 5–8 animals per group [64]. The group size was consistent with established preclinical practices. The experimental unit consisted of a single animal bearing a full-thickness excisional wound, and no animals or data points were excluded from the analysis. To minimize variability, all animals were maintained under identical conditions and subjected to the same experimental procedures. The animals were monitored daily for signs of discomfort or suffering, including skin lesions, facial grimacing, abnormal behavior, and changes in food or water intake, with additional evaluations performed during dressing changes every three days.

The experiment was performed in compliance with the European Community Guidelines (Directive 2010/63/EU) and Romanian legislation (Law no. 43/2014) for the protection of animals used for scientific purposes. The study was approved by the Ethics Committee at Grigore T. Popa University of Medicine and Pharmacy of Iasi (no. 6907/15.04.2020) and by the National Sanitary Veterinary and Food Authority (no. 73/29.07.2024).

##### Wound Model and Protocol Design

The animals were randomly assigned to six experimental groups. General anesthesia was induced by the inhalation of isoflurane (2 L/min), and analgesia was provided via the intraperitoneal administration of tramadol. The dorsal area was carefully shaved and disinfected, and full-thickness excisional wounds (20 mm in diameter) were created using a sterile scalpel. The wounds were positioned approximately 50 mm caudal to the scapulae and 10 mm lateral to the vertebral column. Hemostasis was achieved prior to the applications of the tested materials. CS/PVA and CH/PVA@ACs NF samples (groups 1–4), selected based on the in vitro findings, were applied to the wound sites. Two additional groups served as controls (positive and negative). The experimental design was as follows:

✓ Group 1—CS/PVA NFs;
✓ Group 2—CS/PVA@ARG-ALA NFs;
✓ Group 3—CS/PVA@ARG-RJ NFs;
✓ Group 4—CS/PVA@CUR-ALA NFs;
✓ Group 5—Exufiber^®^, commercial dressing (positive control);
✓ Group 6—Medicomp^®^ sterile gauze (negative control).

The nanofibrous samples were cut into circular patches (20 mm in diameter and 5 mm in thickness) corresponding to an average mass of ~10 mg, ensuring complete coverage of the wound area. The dressings were secured using a click-type fixation device to protect the wound area and prevent displacement by the animals. Dressings were replaced every 3 days over an 18-day experimental period. At each dressing change, the necrotic tissue, fibrin deposits, and excess exudates were carefully removed.

##### Macroscopic Analysis and Planimetric Measurements

The primary outcome measure was the percentage of wound contraction over time. The investigator performing macroscopic evaluation and planimetric analysis using ImageJ software (version 1.54g, National Institutes of Health, Bethesda, MD, USA) was blinded to group allocation. Data were expressed as the mean ± standard deviation (SD). After surgery (day 0), macroscopic evaluation of the wounds was performed at each dressing replacement (days 0, 3, 6, 9, 12, and 15) at the end of the experiment (day 18). At each time point, the wounds were photographed, and the digital images were analyzed using ImageJ software to determine wound size. Wound closure was characterized by the full filling of the defect with regenerated tissue and superficial re-epithelialization. Wound contraction (WC) was determined as the percentage (%) decrease in wound area, using the following formula:

WC (%) = W_0_ − W_t_/W_0_ × 100 (5)

where W_0_ represents the initial wound area (day 0) and W_t_ represents the wound area at different time points.

##### Histopathological Analysis

Full-thickness wound biopsies, along with an extra 5 mm of healthy tissue, were obtained on days 6, 12, 15, and 18. At the end of the experiment (day 18), after the last punch biopsy, the rats were sacrificed by intracardiac administration of 1–2 mL KCl under isoflurane anesthesia. The collected tissue specimens were preserved in 10% formalin, dried using graded ethanol (70–100%), cleaned with xylene, embedded in paraffin, and sectioned at 5 µm for hematoxylin and eosin (H&E) staining and at 4 µm for immunohistochemistry (IHC) examination. Histological analysis was conducted to evaluate wound healing characteristics, including inflammation degree, granulation tissue development, neovascularization, and first connective tissue creation.

IHC labeling was performed with the following primary antibodies: anti-MMP-9 (C-20; sc-6840, mouse monoclonal IgG1), anti-CD31 (ER-MP12; MA1-40074), anti-VEGFA (VG1; MA1-16629), and anti-α-smooth muscle actin (α-SMA; clone 1A4; ab7817). All IHC techniques used positively charged Superfrost™ slides. Following deparaffinization in xylene and rehydration via graded ethanol and distilled water, antigen retrieval was conducted in 10 mM citrate buffer (pH 6) using microwave heating at 95 °C for 10 min, succeeded by a 20-minute cooling period at room temperature. Specimens were then washed twice in PBS for 5 min each and treated with 3% H_2_O_2_ to inhibit endogenous peroxidase activity, followed by two more washes with PBS. For immunohistochemical analysis, IHC blocking was performed using 5% normal serum for 60 min at room temperature prior to the application of the primary antibody. The sections were incubated overnight at 4 °C in a humidified room with primary antibodies at the following dilutions: MMP-9 (1:150), CD31 (1:100), VEGFA (1:100), and α-SMA (1 µg/mL). Following three washes in PBS (5 min each), the slides were treated with the corresponding secondary antibodies. Immunoreactivity was seen with the Novolink™ Polymer Detection System (RE7140K), followed by development with 3,3′-diaminobenzidine (DAB) and counterstaining with hematoxylin. Microscopic evaluation and image acquisition were performed using a Leica DM3000 microscope (Wetzlar, Germany) equipped with a digital camera and Leica Application Suite (LAS) software, version 4.13 for image processing.

### 4.3. Data Analysis

Data are presented as the mean ± SD obtained from at least three independent experiments (n = 3). Statistical analysis was performed using Python (SciPy v1.17, Pandas). The choice of statistical test was dependent on the experimental design. The Student’s *t*-test was applied for pairwise comparisons between the control group (CH/PVA) and bioactive-loaded nanofibrous systems (CH/PVA@ACs), specifically for antioxidant assays (DPPH, ABTS, FRAP, PRAP), hemolysis evaluation, and protein denaturation inhibition. For experiments involving multiple groups and time-dependent analyses, such as cytocompatibility assays (24, 48, and 72 h), one-way analysis of variance (ANOVA) followed by Tukey’s post hoc test (or Bonferroni correction, where appropriate) was used to determine statistically significant differences between groups. Macroscopic wound healing data (wound diameter measurements) were also expressed as the mean ± SD, and statistical comparisons were performed accordingly based on the number of groups analyzed.

## 5. Conclusions

This study supports that CH/PVA NFs functionalized with ACs serve as an effective matrix for wound healing applications. The developed systems demonstrated significant in vitro antioxidant activity, a marked capacity to inhibit protein denaturation, superior hemocompatibility, and minimal cytotoxicity, while in vivo studies validated enhanced tissue remodeling closely approximating normal skin architecture.

The therapeutic efficacy of these materials results from the synergistic interaction of the biocompatible polymeric matrix, the loaded ACs, and the biomimetic nanofibrous structure. Collectively, these attributes provide the concurrent control of many phases of the wound-healing cascade, presenting distinct benefits over traditional single-mechanism treatments.

The in vivo results were equivalent to or superior to those achieved with the commercially available nanofibrous dressing Exufiber^®^, highlighting the significant translational potential of these bioactive-enriched formulations. In acute wounds, the accelerated healing response may shorten recovery time and decrease scar formation, but in chronic wounds, characterized by oxidative stress and ongoing inflammation that hinder repair, the significant antioxidant and anti-inflammatory properties are especially beneficial.

Furthermore, using naturally derived ACs (CUR, ALA, RJ, ARG) in place of synthetic pharmaceuticals may mitigate the hazards linked to prolonged antibiotic treatment and antimicrobial resistance, hence endorsing the appropriateness of these dressings for extended therapeutic uses. This study introduces new opportunities for the strategic design of multifunctional wound dressings that may address several pathophysiological elements of tissue restoration.

## Figures and Tables

**Table 1:** Wound contraction (%) in the CH/PVA@ACs NF-treated groups at different time points.

Group	Sample	Day 3	Day 6	Day 9	Day 12	Day 15	Day 18
1	CH/PVA	24.93 ± 1.60	25.25 ± 1.76	69.40 ± 1.32 *	75.82 ± 1.35 *	78.04 ± 0.01	80.73 ± 0.87
2	CH/PVA@ARG-ALA	5.48 ± 1.86	46.05 ± 2.05	83.52 ± 1.50	97.28 ± 1.75	98.70 ± 0.153	99.91 ± 0.168
3	CH/PVA@ARG-RJ	22.94 ± 1.85	40.18 ± 1.81 *	77.67 ± 1.19 *	95.23 ± 2.19	97.98 ± 0.18	99.94 ± 0.59
4	CH/PVA@CUR-ALA	29.40 ± 1.60 *	34.93 ± 1.55	62.91 ± 1.69 *	89.20 ± 1.72 *	93.86 ± 0.27 *	96.98 ± 0.15
5	Exufiber^®^	15.16 ± 1.92	35.90 ± 1.35	72.14 ± 1.02	94.13 ± 0.62	95.06 ± 0.13	95.06 ± 0.12
6	Medicomp^®^	4.05 ± 2.68	32.40 ± 2.05	66.53 ± 0.59	72.20 ± 1.54	74.72 ± 0.13	76.05 ± 0.95

* *p* ˂ 0.05 when compared to CH/PVA.

## Data Availability

The original contributions presented in this study are included in the article. Further inquiries can be directed to the corresponding author.
